# Extraction of rare earth elements from monazite leach liquor using functionalized chitosan sorbents derived from shrimp waste

**DOI:** 10.1007/s11356-023-29662-8

**Published:** 2023-09-25

**Authors:** Emad A. Matter, Abdelghaffar S. Dhmees, Waheed M. Salem, Mahmoud O. Abd El-Magied, Gaber Hashem Gaber Ahmed

**Affiliations:** 1 Chemistry Department, Faculty of Science, Damanhur University, Damanhur, 22511 Egypt; 2 Analysis and Evaluation Department, Egyptian Petroleum Research Institute, 1 Ahmed El-Zomor St, Nasr City, 11727 Cairo Egypt; 3 Medical Laboratories Department, Faculty of Applied Health Sciences Technology, Menoufia University, Shebin El Kom, 32511 Menoufia Egypt; 4 Nuclear Materials Authority, P.O. Box 530, El Maadi, 11936 Cairo Egypt; 5 Science Department, Rustaq College of Education, University of Technology and Applied Sciences at Rustaq, Rustaq, 318 Sultanate of Oman

**Keywords:** Functionalized chitosan, Lanthanum, Sorption studies, Kinetic, Monazite

## Abstract

With the growing need for high-purity rare-earth elements (REEs), the separation of these REEs has received much attention recently. The objective of this research is to produce chitosan from shrimp waste, then modify it with different functionality, and investigate the adsorption properties of chitosan adsorbents towards La(III) ions. First, from shrimp waste, chitosan (ch) with a significant degree of deacetylation, purity, and solubility was produced. The purified chitosan was cross-linked with epichlorohydrin (ep), and then, it was modified with 3,6,9,12-tetraazatetradecane-1,14-diamine (HA) to produce polyaminated chitosan (HA@ep@Ch). The polycarboxylated/imine chitosan (CM@HA@ep@Ch) was obtained by treating polyaminated chitosan with chloroacetic acid in isopropyl alcohol. The chitosan adsorbents were characterized and applied for lanthanum recovery from synthetic and monazite leach liquor samples. The factors controlling the recovery process were studied and discussed. The performance of the adsorbents was achieved through equilibrium, dynamic, and isothermal studies. HA@ep@Ch and CM@HA@ep@Ch showed good performance for lanthanum recovery with a maximum capacity of 114.52 and 141.76 mg/g at 330 K, respectively. The isotherm parameters refer to the monolayer of lanthanum adsorbed into the adsorbents through chelation and ion exchange mechanisms. A 0.5-M HCl solution was found effective to elute 95.8% of the adsorbed lanthanum on HA@ep@Ch, and 93.4% of the adsorbed lanthanum on CM@HA@ep@Ch. The adsorbents showed greater selectivity in extracting La, Ce, Pr, Nd, and Sm (62–75%) from REE leach liquid compared to extracting other REEs (20–41%).

**Supplementary Information:**

The online version contains supplementary material available at 10.1007/s11356-023-29662-8.

## Introduction

Rare earth elements (REEs) have formed the cornerstone of various technologies and are widely employed in a variety of industries, including contemporary electronics, renewable energy, transportation, health care, and the military. REEs are classified and attributed to variations in their physical and/or chemical characteristics. REEs are classified into two categories, light or LREEs (from La to Eu) and heavy or HREEs (from Gd to Lu) rare earth elements (Chen et al. 2022; Traore et al. 2023). Both LREEs and HREEs can be detected through either primary or secondary sources. There are about 200 minerals categorized as primary or secondary sources because they contain LREEs and/or HREEs (Shahbaz 2022, Talan and Huang 2022). Although there are a great variety and enormous number of REEs minerals (carbonate, silicates, phosphates, etc.), only a few are commercially relevant, namely, bastnaesite (carbonate mineral and its constitution primarily cerium, lanthanum, and yttrium), xenotime (phosphate minerals richer in HREEs), and monazite (phosphate minerals richer in LREEs) (Balaram 2019; Gaete et al. 2021; Shahbaz 2022, Talan and Huang 2022). Secondary sources of REEs are used magnets and catalysts, tailings from lead, iron, zinc, phosphate, sulfur, alumina, and electronic waste. Generally, electronic waste is considered as a potential secondary source of RREs. Also, in secondary sources, LREEs are more prevalent than HREEs. The attractive properties of REEs make them of great importance in different technologies, such as in microelectronics, medicine, catalysts, magnets, mining, and metallurgy. The application of the rare-earth metals (REE) has grown as a result of their diverse uses that benefit critical areas of society. Lanthanum, among the most plentiful and reactive elements between many REEs, has received particular attention. Lanthanum (La) is a diamagnetic, soft, malleable silver white LREE that is commonly found in bastnaesite and monazite. Lanthanum is used in a variety of industries, such as semiconductors, alloys, catalysts, carbon lighting, and additives in glass and ceramic industries (Gaete et al. 2021). The effluents from lanthanum applications are frequently linked with significant amounts of lanthanum waste. As a result, there is potential for recovering lanthanum from these effluents.

REE have comparable chemical and physical characteristics, making it difficult to separate specific elements from primary or secondary sources. Moreover, these sources contain a lower concentration of REE relative to other co-exiting elements. The most popular procedure for REE separations is physical enrichment followed by chemical separations (Fan et al. 2022; Gaete et al. 2021; Traore et al. 2023). Physical separation of REE is based on the differences of the bearing minerals among their physical properties (magnetic, gravity, electrical, etc.). Physical separation is usually initially accomplished by gravity separation, followed by magnetic and electrostatic physical separation methods. Electrostatic separation of REEs depends on the difference in the conductivity of the constituent minerals of the rock sample to be processed, and thus, these constituent minerals can be separated according to their conductivity (Chen et al. 2022; Fan et al. 2022; Traore et al. 2023). Electrostatic separation is frequently utilized when other alternative approaches are ineffective, typically when the constituent minerals have comparable magnetic and specific gravity characteristic. The physically produced REE product is usually processed chemically utilizing various chemical procedures. Froth flotation, precipitation, ion exchange, solvent extraction, sorption, and biosorption are some of the common techniques described for the removal and recovery of lanthanum ions. Flotation is a typical separation process that uses variations in minerals surface wettability caused by interaction with various reagents various surfaces. In flotation, the hydrophobic particles or compounds adhere to air bubbles and float up, leaving the hydrophilic particles or compounds below in the aqueous mixture. Various types of flotation surfactants have been developed to enhance the selective flotation of REE minerals (Chang et al. 2022). Precipitation refers to the formation of insoluble species of the targeted metal ion in solution by adding a reagent to this liquor solution. Acids (i.e., oxalic), bases (hydroxides), and double salts have all been frequently employed to precipitate REE. For the precipitation of REE in the laboratory, oxalic acid is the most commonly used. Precipitation is mostly used to separate REE into two or three fractions and is not regarded an efficient process for obtaining high purity REE (Innocenzi et al. 2015; Nawab et al. 2022). On industrial applications, liquid–liquid extraction, solvent extraction, has been widely employed for the separation of several elements, including REE. Liquid–liquid extraction is an effective, efficient, simple, and fast separation process. Different organic solvents have been used for liquid–liquid extraction of lanthanum, such as phosphorus, carboxylic acids, and amine solvents (Innocenzi et al. 2015, Nawab et al. 2022, Pattanaik and Mishra 2022). The most challenging problem with the liquid–liquid method is that the used organic solvents are volatile, flammable, and carcinogenic, which leads to safety, economic, and environmental concerns resulting from increasing consumption, cost, and health issues.

Among all the separation processes for REE removal and recovery, solid/liquid separation technology, i.e., ion exchange, adsorption, and biosorption methods, has played a significant role in the separation of different metal ions. Solid/liquid separation has the advantages of efficiency, simplicity, selectivity, eco-friendliness, and economy, particularly for lanthanum recovery from various effluents (Wang et al. 2023; Yuhua et al. 2022). The ion exchange separation method is based on the exchange of similar-ions (or molecules) of similar charge between the liquid phase and the contact solid phase. Different applications that include medicine, food, hydrometallurgy, and oil sectors have all used ion exchange systems. Many ion exchange resins have been utilized for REE separation (Lankapati et al. 2021; Ni et al. 2021). Biosorption has been proposed as an option for the separation of REE because of its cost, efficiency, and simplicity in REE separation from low concentrations effluents. Recently, different biosorbent materials have been used as efficient adsorbents for recovering REE from different leach solutions (electronic waste, monazite…, etc.) (Banu et al. 2020; dos Reis et al. 2022; Li et al. 2020). The mechanism of REE biosorption is influenced by the kinds of biosorbent, solution chemistry (of biosorbent and of metal), and ambient/environmental variables (i.e., pH). Recently, different biosorbent materials, such as chitosan, clay, and cellulose (Billah et al. 2022, Khapre and Jugade 2020, Korde et al. 2021, Korde et al. 2022, Makhado et al. 2022, Shekhawat et al. 2017), have been used as efficient adsorbents for recovering REEs and other metal ions from leach solutions (Banu et al. 2020; dos Reis et al. 2022; Li et al. 2020). In addition, shrimp is among the most important marine products in the world. The head and skin of the shrimp are separated before packing for use or export. Therefore, the shrimp heads and skins are considered a waste by that industry. This shrimp waste has very low economic value (it can be used in the animal feed industry). Shrimp and seafood industries produce enormous amounts of huge quantities of shrimp offal, about 50.0–55.0% of the shrimp weight. Chitin is a β-D-(acetylamino)-2-deoxy-glucopyranose linear chain polymer (No et al. 1989). Each β-D-(acetylamino)-2-deoxy-glucopyranose residue rotates 180° from the previous β-D-(acetylamino)-2-deoxy-glucopyranose residue. The high tensile strength of chitin is due to the hydrogen bonding between β-D-(acetylamino)-2-deoxy-glucopyranose residues. Chitin is classified into two categories, alpha and beta, and can be distinguished by its content of hydrogen bonds. Alpha type possesses hydrogen bonds that link the alternating poly-saccharide chains in its structure, while beta type does not contain these hydrogen bonds. Also, alpha type is the more stable than beta type, and therefore more resistant to deacetylation than beta type.

Chitosan is a non-toxic and biodegradable carbohydrate polymer derived from chitin by alkaline deacetylation or by enzymatic degradation (El Knidri et al. 2018; Sunilkumar et al. 2014). Chitosan is a deacetylchitin (or poly-(D)glucosamine natural polymer. This natural deacetylchitin polymer is composed much like cellulose polymer with a sugar backbone with β-1,4-linked D-glucosamine; the one difference being the (-OH) group in the C-2 position is substituted by (-NHCOCH_3_) group (Sunilkumar et al. 2014). The acetyl concentration of the polymer distinguishes chitin from chitosan. Also, chitosan dissolves in water and in most dilute acids (due to its hydrophillicity), while cellulose is not. The amino (NH_2_) groups on chitosan serve as ion exchanger sites and chelation sites for heavy metal ions. Due to chitosan’s poor mechanical characteristics, chemical and physical modifications (cross-linking) are necessary (El Knidri et al. 2018). Chitosan has positive ionic charges (at pH below 6.3). Chitosan has gained increasing commercial interest because of its distinctive properties such as biodegradability, ease of modification, biocompatibility, and ability to interact with different adsorbates. The main functional groups of chitosan are the -NH_2_ group (positioned at C-2), the primary -OH group (positioned at C-6), and the secondary -OH group (positioned at C-3). Changing of modifying these groups (-NH_2_ groups, primary -OH groups, and/or secondary -OH groups) changes the properties of the molecule to make it suitable for specific applications (El Knidri et al. 2018; No et al. 1989). Modifying or changing chitosan structure results in a significant change in the properties of the resulting molecule (modified chitosan molecule). Chitosan-based composites have attracted great attention in various applications due to their impressive properties such as mechanical strength, chemical stability, surface area, and structural properties (Abdulhameed et al. 2022b, 2022c; Jawad et al. 2022a, 2023; Rosli et al. 2023; Zain et al. 2023). In this study, chitosan (Ch) with a significant degree of deacetylation, purity, and solubility was produced from shrimp waste, and then, it was modified with NH_2_ and COOH ligands to produce different functionalized cross-linked chitosan/poly-amine (HA@ep@Ch) and poly carboxylated/imine chitosan (CM@HA@ep@Ch) biosorbents. The recovery of lanthanum by the modified chitosan adsorbents was thoroughly explored. The influence of several factors on La(III) ion adsorption by the prepared adsorbents was tested. The tested adsorption factors include pH, La(III) concentration (*C*, mg/L), duration (*t*, min), and temperature (*T*, K). Also, La(III) separation from monazite leach was investigated, as well as the adsorption mechanism.

## Materials and methods

### Chemicals

Fresh shrimp waste was collected from a local market. HCl (37%), HNO_3_ (68–70%), NaOH (≥ 97%), ethanol (≥ 99%), La(NO_3_)_3_·6H_2_O (≥ 99%, as a source of La(III)), and 3,6,9,12-tetraazatetradecane-1,14-diamine (HA) were Sigma-Aldrich products. Epichlorohydrin (ep) and isopropyl alcohol were Sigma-Aldrich products. All the solutions used in this work were prepared using double distilled water (DDW). The solution pH was adapted with a 0.01-N solution of NaOH and/or HCl. La(III) ions were detected by a UV–visible spectrophotometer using the Arsenazo III dye (Marczenko 1975).

### Chitosan extraction

Fresh shrimp waste was collected from a local market. After being rinsed with tap water, the shrimp waste was dried (300 K for 8 h) and crushed. The crushed shrimp waste was subjected to a demineralization process (Abd El-Magied 2023). The crushed shrimp waste (1000 g) was demineralized for 24 h using 2.0N HCl at 318 K. The waste (wt., g) to HCl (*v*, mL) ratio was 1: 15 (wt.: *v*). The demineralized material was filtered and thoroughly washed with DDW (until neutral pH) before being dried at 340 K. The demineralized shrimp waste (588.6 g) was deproteinized for 24 h at 300 K with 5% NaOH. The demineralized shrimp waste was added to NaOH solution with ratio of 1:5 (wt.: *v*). The residue (chitin) was rinsed in DDW until it reached a pH level of neutrality. The obtained chitin was then dried and crushed and then subjected to chitosan. The obtained chitin product (273.7 g) was treated with NaOH (50%) with a chitin/NaOH ratio of 1:12 (w/v). The suspension was stirred at 340 K for 24 h. The residue (chitosan) was washed until neutral pH with hot DDW. The obtained chitosan was then dried at 340 K for 6 h. The yield of chitosan was 211 g.

### Synthesis of aminated (HA@ep@Ch) and carboxylated cross-linked chitosan and (CM@HA@ep@Ch)

Chitosan powder (2.5 g) was dissolved in 100.0 mL of acetic acid solution (5%) under stirring (for 24 h; at 300 K) to form a clear chitosan solution (Abd El-Magied 2023). Then, the chitosan content was precipitated into 1 L NaOH (2 M) containing 10% ethyl alcohol. Chitosan gel was filtered and washed well with distilled H_2_O (to pH about 7) to obtain the purified chitosan product. The obtained purified chitosan was added to 100 mL NaOH (pH 10), stirring at room temperature for 15 min. Add 70 mL isopropyl alcohol to the former solution and stirring at room temperature for another 20 min, and then, add 20 mL epichlorohydrin drop wise over 30 min. Check pH and adjust it to 10–12. The mixture was agitated under gentle heating until the gelatinous product (crosslinked chitosan) was obtained (be careful about pH to be at 10–12). Filter and wash the product well with distilled H_2_O, and then, wash with 0.1 M NaOH. Wash the product again with 0.1 M HCl followed by distilled H_2_O. The obtained product (crosslinked chitosan, ep@Ch) was insoluble in acetic acid. The crosslinked chitosan (ep@Ch) was added to 50 mL isopropanol alcohol (under stirring for 15 min). The cross-linked epichlorohydrin/chitosan (Cl@ep@Ch) was prepared by adding 2.5 mL of epichlorohydrin solution (25%) to the former chitosan solution (drop wise addition). After stirring for 30 min, heat at the mixture at 333 K for 2 h. The obtained Cl@ep@Ch was washed several times with ethanol followed by DDW. The aminated cross-linked chitosan (HA@ep@Ch) was produced by the direct interaction between Cl@ep@Ch and 3,6,9,12-tetraazatetradecane-1,14-diamine (HA) in an alcoholic medium. 3,6,9,12-Tetraazatetradecane-1,14-diamine (HA; 4.0 mL) was added drop wise to 32.0 mL of DMF with stirring at 300 K. The mixture was gently stirred for 20.0 min, and then, 1.0 g of Cl@ep@Ch was added portionwise under stirring. At 300 K, the reaction mixture was agitated for 30 min, after which the temperature was raised to 370 K. After stirring for 6 h, the solvent (DMF and unreacted HA) was eliminated by filtration of the reaction mixture. The obtained solid product (HA@ep@Ch) was washed several times with ethanol and DDW and finally dried at 345 K for 24 h. In 30.2 mL of DMF/water solution (2:1), 1.0 g of potassium chloroacetate was dissolved. The obtained solution was agitated for 20.0 min at 300 K, after which HA@ep@Ch (1.0 g) was added portionwise to the reactor solution. After stirring for 20.0 min, the temperature of the reactor was raised to 378 K. After stirring for 6 h, the solvent was eliminated by filtration of the reaction mixture. The solid product (CM@HA@ep@Ch) obtained was washed several times with ethanol and DDW before being dried at 345 K for 24 h. The amine group content of the HA@ep@Ch was estimated by soaking 1.0 g of the HA@ep@Ch in 100.0 mL of 0.1 M HCl at 300 K for 24 h. Afterwards, the HCl solution was eliminated by filtration of the reaction mixture. The concentration of HCl in the filtrate was determined by titration with stander solution of NaOH (0.1 M). The amine group content in the HA@ep@Ch equals the difference between initial (0.1 M) and final HCl concentrations (the concentration of HCl in the filtrate). The carboxylic group content of the CM@HA@ep@Ch was estimated by soaking 1.0 g of CM@HA@ep@Ch in 100 mL of 0.1 M NaOH at 300 K for 24 h. Afterwards, the NaOH solution was eliminated by filtration of the reaction mixture. The remaining NaOH concentration (in the filtrate) was determined by titration with stander HCl solution (0.1 M). The carboxylic group content in the CM@HA@ep@Ch equals the difference between initial (0.1 M) and final NaOH concentrations (the concentration of NaOH in the filtrate).

### La(III) biosorption on HA@ep@Ch and CM@HA@ep@Ch

The pH effect on the biosorption of La(III) by the HA@ep@Ch and CM@HA@ep@Ch adsorbents was tested in the range of 1.0–7.0. The biosorbent (0.025 g; ep@Ch, HA@ep@Ch and CM@HA@ep@Ch) was added to 100.0 mL round bottles. Then, 50.0 mL of 150.0 mg/L La(III) ion solution at different pH values was added to it. The bottles were shacked using a water bath shaker at 300.0 rpm. After 180 min, the biosorbents were filtrated, and the La(III) ions were determined in the filtrate (Marczenko 1975). The biosorbent capacities (*q*_*e*_, mg/g) were calculated from the difference between the initial La(III) concentration (*C*_*i*_, mg/L) and the final or equilibrium La(II) concentration (*C*_*e*_, mg/L) against the biosorbent mass (*Wt*, mg/g) using Eq. 1. By using the same data, the biosorbention efficiency (*q*_*e*_, %) was calculated (Eq. 2) (Abd El-Magied et al. 2021a).

1 $${q}_{e} (\mathrm{mg}/\mathrm{g})=\frac{\left({C}_{0}-{C}_{e})\times (V\right)}{Wt}$$

2 $${q}_{e} (\mathrm{\%})=\frac{\left({C}_{0}-{C}_{e})\times (100\right)}{{C}_{0}}$$

where *V* is the volume of La(II) solution (L). The effect of time (10.0–180.0 min) was studied at 300 K using the same procedure. In 100.0 mL round bottles containing 50.0 mL of a 150.0-mg/L La(III) ion solution at pH 6.0, 0.025 g of the biosorbent (HA@ep@Ch or CM@HA@ep@Ch) was added. The bottles were shacked using a water bath shaker at 300.0 rpm for the required time. The HA@ep@Ch and CM@HA@ep@Ch were filtrated, and the La(III) ions were determined in the filtrate. The biosorption capacities obtained at different times were plotted against time for different kinetic studies. At temperatures ranging from 300 to 330 K, the effect of initial La(III) concentrations (10.0–400.0 mg/L) was investigated. The biosorbent (0.025 g) was added to 50.0 mL of the La(III) solution at pH 6.0. The bottles were shaken at the required temperature for 120 min. The biosorption capacities obtained at different concentrations and temperatures were plotted against equilibrium concentrations for different isotherm studies. The effect of biosorbent mass/liquid La(III) solution (solid/liquid ratio, S/L) was studied at optimum conditions obtained from previous experiments, while the biosorbent mass was varied. Different biosorbents (0.025–0.5 g) were added to 50.0 mL of the La(III) solution (400 mg/L) at pH 6.0. The bottles were shaken at 300 K for 120 min. The biosorption capacities (mg/g) and biosorption efficiencies (mg/g) obtained were plotted against S/L.

### Elution of La (III) and biosorbent regeneration

The elution of La(III) from the loaded biosorbents was studied using different concentrations of hydrochloric acid (from 0.01 M to 0.5 M HCl). For 24 h, 0.025 g of the La (III)-loaded biosorbent was stirred in 50.0 mL of HCl solution (0.01–0.5 M). The eluted La(III) ions (*C*_Elution_) were analyzed in the elution medium. Using Eq. 3, the elution parentage (%) was calculated as the difference between adsorbed ((*C*_*i*_ − *C*_*e*_)_adsorption_) and eluted La (III) ions (*C*_Elution_).

3 $$\mathrm{Elution }(\mathrm{\%})=\frac{100\times {C}_{\mathrm{Elution}}}{{(C}_{0}-{C}_{e}{)}_{\mathrm{Adsorption}}}$$

The biosorbent reusability was evaluated through three consecutive adsorption/elution cycles. In each cycle, 0.025 g of the biosorbent was stirred into 50.0 mL of La(III) solution (400 mg/L, pH 6.0) for 120 min. The La(III) ions (C_e_) were analyzed in the filtrate medium. Then, the resultant La(III)/bio sorbent was stirred in 50.0 mL of HCl solution (0.4 M) for 24 h. The eluted La(III) ions (*C*_Elution_) were analyzed in the elution medium. Prior to the next adsorption/elution cycle, the eluted biosorbent was washed thoroughly with DDW to pH = 7. The biosorbent reusability (Reg. effic., %) was computed from the biosorption capacity differences between the first and second cycle, Eq. 4 (Abd El-Magied et al. 2021a).

4 $$\mathrm{Reg}. \left(\mathrm{\%}\right)=100\times \frac{\mathrm{ Sorption capacity of the adsorbent }(\mathrm{seond cycle})}{\mathrm{ Biosorption capacity of the adsorbent }(\mathrm{first cycle})}$$

## Results and discussions

### Characterization of the adsorbents

**Fig. 1:**
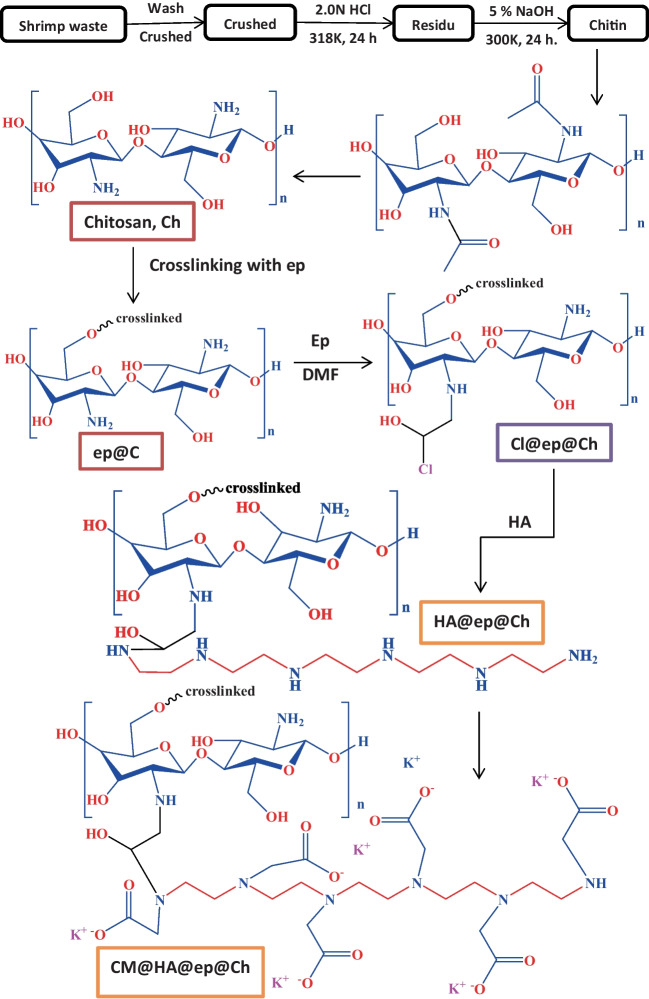
Synthesis route of Ch, ep@Ch, HA@ep@Ch, and CM@HA@ep@Ch

Figure 1 shows the synthesis of HA@ep@Ch and CM@HA@ep@Ch from shrimp waste. The different characteristics of shrimp, chitin, chitosan, ep@Ch, HA@ep@Ch, and CM@HA@ep@Ch samples were analyzed by different analytical methods (Abd El-Magied 2023). The yield of chitin extracted from shrimp waste is 22.1%. The yield of the shrimp chitosan is 18.01. Shrimp waste, chitin, and chitosan have moisture contents of 68.5, 8.41, and 9.66%, respectively. The increase in moisture content of chitosan relative to that of chitin may be due to the hygroscopic properties of chitosan. ep@Ch, HA@ep@Ch, and CM@HA@ep@Ch have moisture contents of 9.21, 9.95, and 9.15, respectively. The ash content of chitosan (or chitin) is an indication of the efficacy of the demineralization of the shrimp waste (the lower the ash content in the final product, the more effective the demineralization process). The residual calcium carbonate in the final shrimp chitosan product affects its characteristics (i.e., decreases its solubility and viscosity). Generally, it is necessary to obtain a chitosan product that has a < 1% ash content (Sunilkumar et al. 2014). The shrimp waste had a 31.1% ash content. Demineralization elimination resulted in a chitin product having a 0.27% ash content. In this study, the obtained shrimp chitosan had 0.21% ash content. ep@Ch, HA@ep@Ch, and CM@HA@ep@Ch have ash contents of 0.22, 0.23, and 0.24, respectively.

**Fig. 2:**
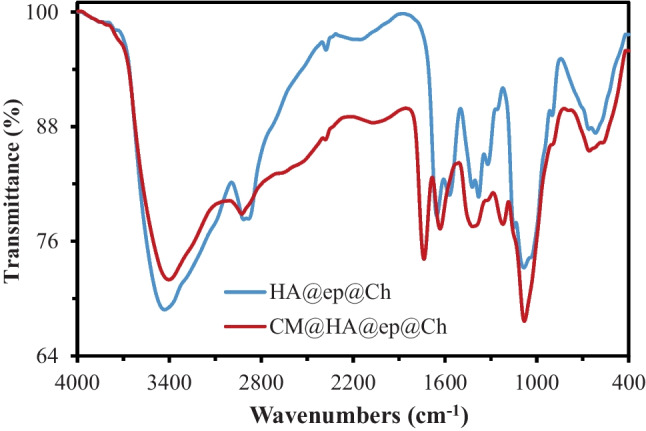
FT-IR spectra of HA@ep@Ch and CM@HA@ep@Ch adsorbents

**Fig. 3:**
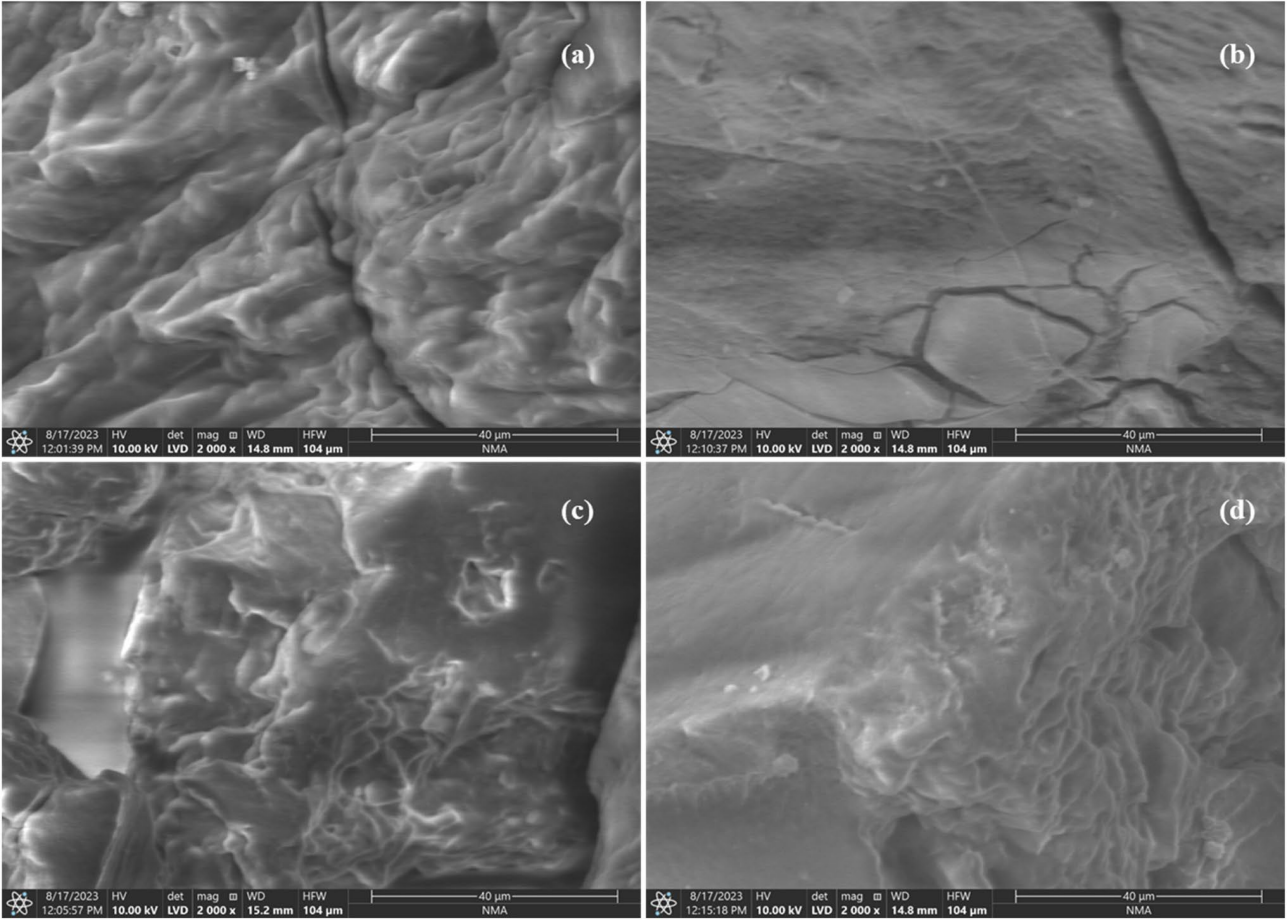
SEM images of HA@ep@Ch, La (III)-loaded HA@ep@Ch, CM@HA@ep@Ch, and La (III)-loaded CM@HA@ep@Ch sorbents

**Fig. 4:**
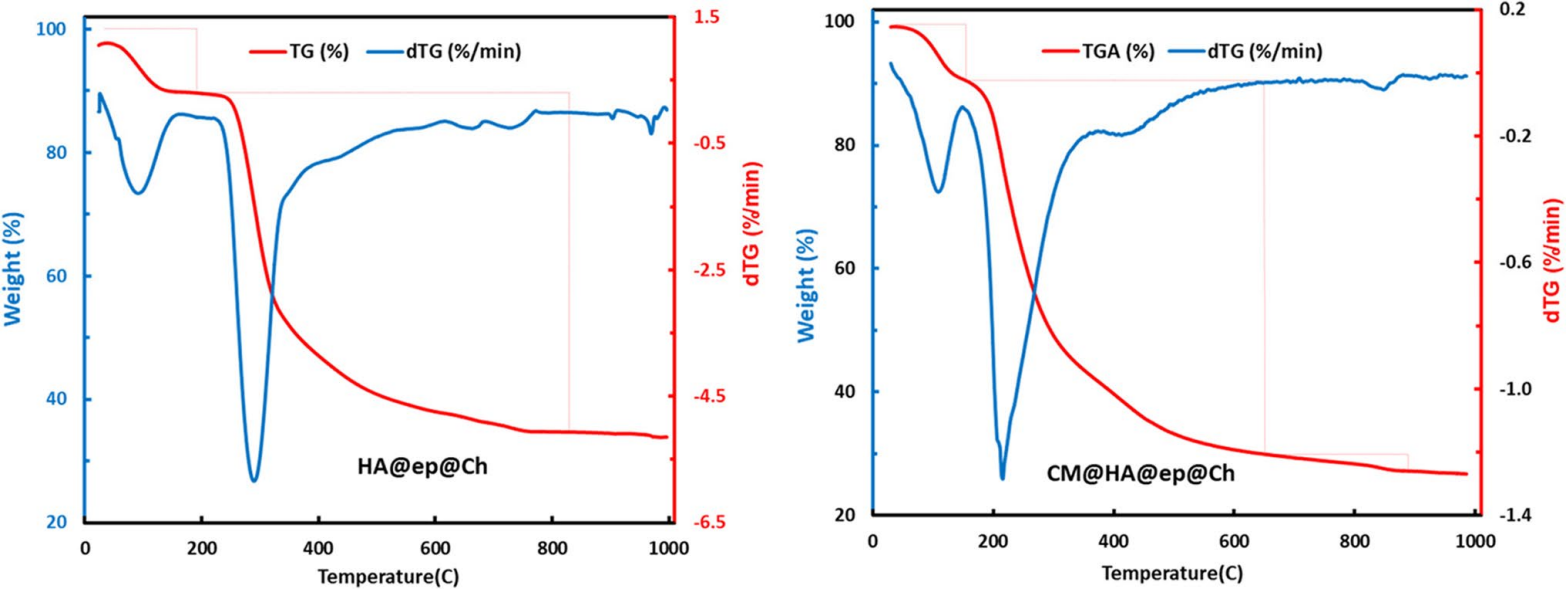
Thermogravimetric analysis (TG (%) and dTG (%/min) of HA@ep@Ch and CM@HA@ep@Ch biosorbents

The FT-IR spectra of HA@ep@Ch and CM@HA@ep@Ch are shown in Fig. 2. For HA@ep@Ch, the spectra contains a strong and broad band at 3419.61 cm^−1^ related to the OH groups, band at 3307.73 related primary and to secondary aliphatic NH groups, band at 2908.49 related to C = CH_2_ groups, band at 2875.71 related to C-H, band at 2140.86 related to C-N–C, band at 1643.26 related to substituted alkenes, band at 1566.11 related to primary amines, band at 1429.17 related to CH (bending), band at 1390.61 related to methylene, band at 1325.02 related to OH (bending), band at 1128.29 related to C-N of amine, band at 1082.01 related to C-N, band at 1035.72 aliphatic amines, band at 900.71 related to C = CH_2_), and bands at 655.76 and 617.19 related to substituted C = C (bending) (Movasaghi et al. 2008; Yu et al. 2017). The presence of the characteristic bands of amino groups (3307.73 2140.86, 1566.11, 1128.29, 1082.01, and 1035.72) indicates the successful synthesis of cross-linked chitosan/poly-amine adsorbent (HA@ep@Ch). The FT-IR spectra of CM@HA@ep@Ch contain bands at 3425.38 (OH groups), 3257.58 (secondary NH groups), 2914.27 (C = CH_2_), 1728.12 (carboxylic acids, non-ionized), 1629.76 (C = O, carboxylates), 1398.32 (methylene), 1215.08 (C-O of carboxylic acids), 1082.03 (C-O of alcohols and C-N), 894.92 (C = CH_2_), and 655.76 (C = C, bending) (Movasaghi et al. 2008; Yu et al. 2017). The presence of the characteristic bands of COO groups (1728.12, 1629.76, 1215.08, and 1082.03) indicates the successful synthesis of poly carboxylated/imine chitosan adsorbent (CM@HA@ep@Ch). Additionally, the noticeable changes on the characteristic bands of amino groups (of CM@HA@ep@Ch relative to that of HA@ep@Ch) also indicate the successful synthesis of poly carboxylated/imine chitosan adsorbent (CM@HA@ep@Ch). The FT-IR spectra of HA@ep@Ch and CM@HA@ep@Ch after La(III) adsorption show many changes in the wavenumber and intensities of the function groups caused by La(III) adsorption (Supplementary information, SI, Fig. S1). The surface area and the pore distribution were calculated by Brunauer–Emmett–Teller (BET) and Barret-Joyner-Halenda (BJH) equations at 77 ± 1 K by a Quanta Chrome NOVA automated gas sorption system using N_2_ gas as the adsorbate. The surface area of the HA@ep@Ch and CM@HA@ep@Ch was 45.63 and 29.64 m^2^/g, respectively. After lanthanum adsorption, the surface area of the loaded HA@ep@Ch and CM@HA@ep@Ch adsorbents was 21.32, and 16.15 m^2^/g, respectively. The surface morphologies of HA@ep@Ch and CM@HA@ep@Ch products were analyzed by SEM and EDX analysis. The surface features of HA@ep@Ch and CM@HA@ep@Ch were examined using the scanning electronic microscope with an energy-dispersive X-ray spectrometer (SEM/EDX) (model XL 30 ESEM). The SEM images and EDX charts of HA@ep@Ch and CM@HA@ep@Ch products are presented in Fig. 3 and Fig. S2 (SI). The SEM images show changes in the surface texture before and after sorption of lanthanum ions (Fig. 3). The EDX charts of HA@ep@Ch and CM@HA@ep@Ch confirm their elemental composition of C, H, O, and N atoms (Fig. S2). Thermogravimetric analysis (TG (%) and dTG (%/min) of HA@ep@Ch and CM@HA@ep@Ch biosorbents was utilized to obtain quantitative information on weight losses as a function of time and temperature in conjunction with a mass spectrometry analysis (Fig. 4). The TGA curves (TG (%)) of HA@ep@Ch and CM@HA@ep@Ch represent a multi-stage decomposition at 35–889 °C. For HA@ep@Ch, there are two stages of decomposition occurred at temperature range of 35–191 °C (mass loss of 8.1%) and 228–829 °C (mass loss of 54.6%). There were three stages of decomposition for CM@HA@ep@Ch, which occurred at temperatures ranging from 38 to 154 °C (mass loss of 9.5%), 157 to 650 °C (mass loss of 59.4%), and 814–889 °C (mass loss of 2.7%). These thermogravimetric results show that CM@HA@ep@Ch biosorbent is relatively more stable than HA@ep@Ch biosorbent. On the other hand, the thermogravimetric analysis (dTG (%/min)) of HA@ep@Ch and CM@HA@ep@Ch biosorbents shows that all peaks are endothermic peaks. These endothermic peaks are related to the decomposition stages of HA@ep@Ch and CM@HA@ep@Ch.

### Effect of pH

**Fig. 5:**
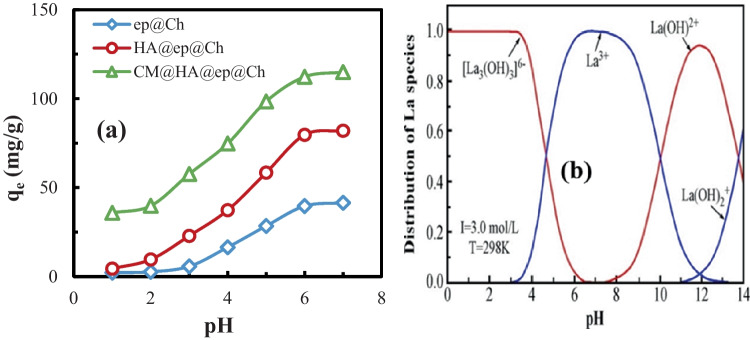
Effect of pH (**a**) and La(III) species at various pH values (**b**)

The effect of pH (1.0–7.0) and ionic strength (0.001–0.1 M NaCl) on the biosorption of La(III) ions on the biosorbents was studied in synthetic La(III) solution (Fig. 5a). The results obtained indicate that the quantity of adsorption rises as the pH of the medium rises until the medium pH reaches 6, after which the increase in the amount of adsorption is almost imperceptible and can be ignored. This behavior may be related to the sensitivity of the surface groups of the biosorbent (NH_2_, NH, OH, and COOH) to the pH of the medium. The lower biosorption capacity at low pH may be related to the electrostatic interaction between the surface groups of the biosorbent (NH_2_, NH, OH, and COOH) and the hydrogen protons (H^+^) of the adsorption medium. This electrostatic interaction resulted in protonated biosorbent surface groups (NH_2_, NH, OH, and COOH) which cannot adsorb La(III) from the adsorption medium due to electrostatic repulsion between the biosorbent and the adsorbate. Figure 5 b shows the proportion of La (III) species as an attribute of various pH values. The major La(III) species at very low pH (around pH 1.0) La_5_(OH)_9_^6+^ and the considerable quantity of H^+^ present at this pH value can compete with the La_5_(OH)_9_^6+^ adsorption, resulting in poor lanthanum adsorption. Also, the greater ionic radius of La_5_(OH)_9_^6+^ relative to H^+^ makes La(III) adsorption harder. At pH > 1.0, the predominant lanthanum species is La^3+^. La^3+^ ions have small ionic radius (relative to La_5_(OH)_9_^6+^), so La(III) species are more readily adsorbed than La_5_(OH)_9_^6+^ (the diffusion of La(III) ions into the biosorbents is easier than for La_5_(OH)_9_^6+^ species). In addition, as pH increases, the biosorbent protonation weakens; the carboxylate groups of the biosorbent exist mainly as ionized COO^−^. Also, amino groups become ionized, and thus, affinity towards La(III) increases, and thus, the amount of La(II) loading is increased. Therefore, the optimum pH of the medium used in the rest of the extraction experiments is 6. The suggested mode of interaction between La(III) species and the biosorbents is a complexation reaction (Eqs. 5 and 6) and cation exchange reaction (Eq. 7).

5 $$\mathrm{Modified chitosan }\cdots {\mathrm{NH}}_{2}+{\mathrm{La}}^{3+}\to [\mathrm{Modified chitosan}\cdots {\mathrm{NH}}_{2}\mathrm{La}{]}^{3+}$$

6 $$\mathrm{Modified chitosan}\cdots \mathrm{COOH}+{\mathrm{La}}^{3+}\to [\mathrm{Modified chitosan}\cdots \mathrm{COOHLa}{]}^{3+}$$

7 $$\mathrm{Modified chitosan}\cdots {\mathrm{COO}}^{-}{\mathrm{H}}^{+}+{\mathrm{La}}^{3+}\to (\mathrm{Modified chitosan}\cdots \mathrm{COO}{)}_{3}\mathrm{La}+3{\mathrm{H}}^{+}$$

Sodium chloride was to the adsorption medium to investigate the influence of ionic strength on adsorption (SI, Fig. S3). The concentration of additional sodium chloride ranged from 0.01 to 0.1 M, and the other parameters were the same as in the previous pH experiments. As expected, increasing NaCl amount on the adsorption medium decreased the ability of the adsorbents to absorb lanthanum from the medium. This behavior refers to the outer sphere adsorption mechanism. This finding might be explained by competing adsorption between Na^+^ and La(III) ions for the same adsorbent active sites.

### Bisorption kinetics

**Fig. 6:**
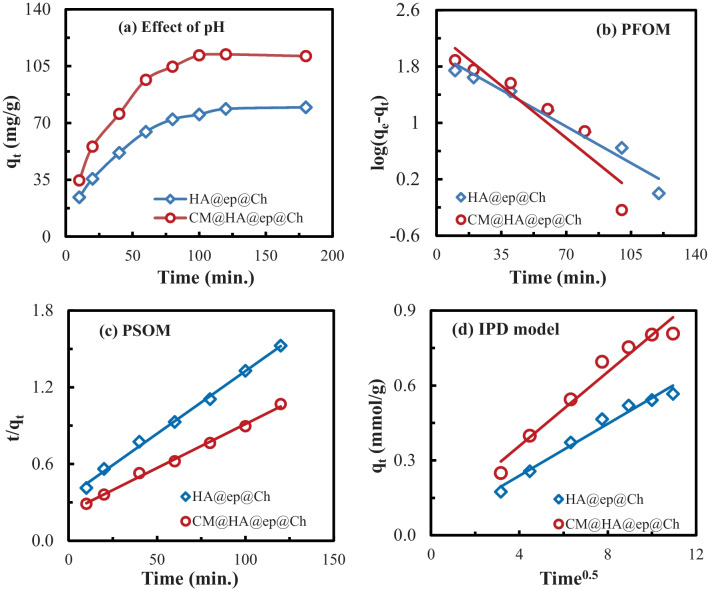
Bisorption kinetics profile for La(III) adsorption on HA@ep@Ch and CM@HA@ep@Ch biosorbents

Figure 6 a depicts the influence of time on the HA@ep@Ch biosorption of La (III) ions. The results obtained indicate that the amount of adsorption increases with the increase in the contact time (the sorption rate increased rapidly) until the time reaches 60 min. After 60 min, the adsorption rate increased slowly till equilibrium was attained (120 min), after which the increase in the amount of adsorption is almost imperceptible and can be ignored. This behavior may be related to the availability of vacant binding sites on the biosorbents. This availability decreases with time, which results in a reduced adsorption rate. So, as time progressed, the reaction between the HA@ep@Ch or CM@HA@ep@Ch biosorbents and La(III) ions led to a decrease in the vacant binding sites; hence, the adsorption was reduced. Therefore, the optimum time used in the rest of the extraction experiments is 120 min.

The biosorption order and rate of La (III) adsorption onto the HA@ep@Ch and CM@HA@ep@Ch biosorbents were described using pseudo first (PFOM) (Fig. 6b), pseudo second (PSOM) (Fig. 6c), and intraparticle diffusion (IPD) equations (Fig. 6d) (Abd El-Magied et al. 2021b, Abd El-Magied 2016). The outcomes of the used kinetic models were used to predict biosorption mechanism and rate-controlling steps. Table 1 lists the used kinetic models and their outcomes. The high correlation coefficients (*R*^2^ > 0.996) in Table 1 suggest that the PSOM matches the experimental data better than the PFOM (*R*^2^ < 0.961); accordingly, the biosorption of La (III) onto the HA@ep@Ch and CM@HA@ep@Ch biosorbents occurs via a chemisorption route. According to the intraparticle diffusion hypothesis (or Weber-Morris model), adsorption varies approximately proportionately with (*t*^0.5^) rather than with time (*t*). The outcomes of the Weber-Morris model were revealed from the intraparticle diffusion model plots (Fig. 6d). The values of *C* (0.0306 and 0.0611) and *K*_*ip*_ (0.052 and 0.0741) were small for both ch@ep@PA and ch@ep@PA@CM biosorbents, respectively. In addition, the relative higher *R*^2^ values (0.9749 and 0.9611) refer to an adsorption process of La(III) controlled by the intraparticle diffusion mechanism. So, this study demonstrates that the La(III) uptake on HA@ep@Ch and CM@HA@ep@Ch is dictated by pore-diffusion control. The overall kinetic studies refer to a chemical interaction between La(III) and the used biosorbents (HA@ep@Ch and CM@HA@ep@Ch) controlled by pseudo-second-order kinetics and a pore-diffusion mechanism.

**Table 1:** A summary of the kinetic models used and their outcomes

Model	Linear equation	Plot	
PFOM	$\log \left({\textrm{q}}_{\textrm{e}}-{\textrm{q}}_{\textrm{t}}\right)=\log \left({\textrm{q}}_{1\textrm{st}}\right)-\frac{{\textrm{k}}_1}{2.303}\textrm{t}$	Log(q_e_-q_t_) vs. t	
parameters	Defination	HA@ep@Ch	CM@HA@ep@Ch
q_t_ (mg/g)	Experimental capacity	79.65	112.23
q_1st_, (mg/g)	PFOM capacity	96.69	186.55
k_1_, (min^-1^)	PFOM rate constant	0.0341	0.4882
R^2^	Correlation coefficient	0.9616	0.8896
PSOM	$\frac{\textrm{t}}{{\textrm{q}}_{\textrm{t}}}=\frac{1}{{\textrm{k}}_2{\textrm{q}}_{2\textrm{nd}}^2}+\frac{1}{{\textrm{q}}_{\textrm{e}}}\textrm{t}$	t/q_t_ vs. t	
parameters	Defination	HA@ep@Ch	CM@HA@ep@Ch
q_2nd_, (mg/g)	PSOM capacity	102.04	144.92
k_2_, (g/mg.min.)	PSOM rate constant	0.0003	0.0002
R^2^	Correlation coefficient	0.9967	0.9961
IPD	q_t_ = k_id_t^0.5^ + C	q_t_ vs. t^0.5^	
parameters	Defination	HA@ep@Ch	CM@HA@ep@Ch
K_id_, (mg/g.min^0.5^)	IPD rate constant	0.0520	0.0741
C	IPD constant proportional	0.0306	0.0611
R^2^	Correlation coefficient	0.9749	0.9611

### Effect of adsorbent and adsorbate dose

**Fig. 7:**
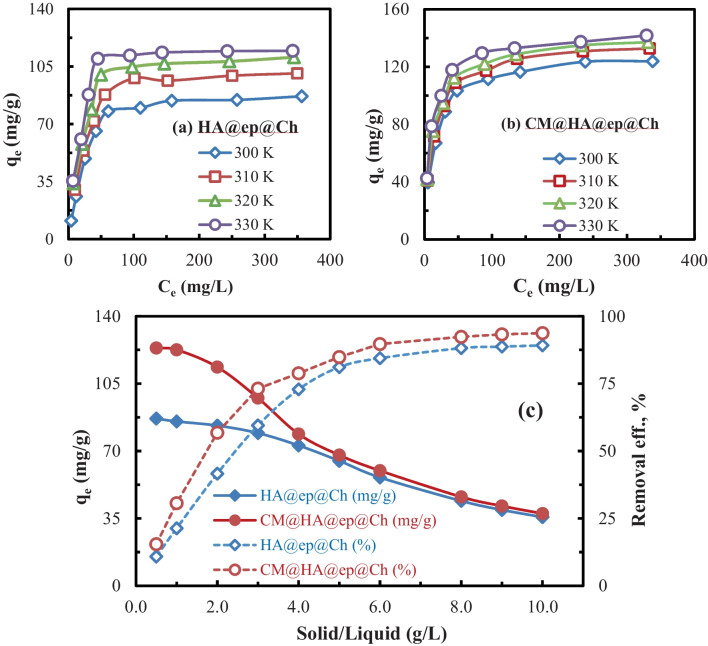
Effect of La (III) concentration (**a**, **b**) and adsorbent dose (**c**) on HA@ep@Ch and CM@HA@ep@Ch biosorption capacity and efficiency

The effect of the initial concentration of La(III) ions (adsorbate dose) on the adsorption is shown in Fig. 7 a and b. The biosorption capacities are increased gradually as the La(III) concentration increases until equilibrium is attained (300 mg/L). The increase in the biosorption capacities with increasing La(III) initial concentration may be related to increasing the collision probability between La(III) ions and the HA@ep@Ch or CM@HA@ep@Ch surfaces (Abdulhameed et al. 2022a; Jawad et al. 2022b; Suhaimi et al. 2022). The collision probability between La(III) ions and the biosorbent surface at higher La(III) concentrations are increased due to the La(III) ions availability for the same binding sites at the HA@ep@Ch or CM@HA@ep@Ch surface. So, as the concentration of La(III) increased, (i) the collision probability between La(III) ions and the HA@ep@Ch or CM@HA@ep@Ch surfaces increased, (ii) the availability of La(III) ions for the same binding sites at the HA@ep@Ch or CM@HA@ep@Ch surfaces increased, and (iii) the resistance to mass transfer of La(III) from the adsorption medium to the adsorbent surface decreased. These effects resulted in enhanced La(III) adsorption at higher La(III) initial concentration.

The previous experiment (effect of La(III) initial concentration) was done at a different temperature (300–330 K) to investigate the effect of temperature on the La(III) biosorption process. Figure 7 a and b show an enhancement in biosorption capacities at higher temperatures, referring to the endothermic nature of La(III) biosorption on the HA@ep@Ch and CM@HA@ep@Ch. The biosorption capacities obtained at higher temperatures may be due to (i) the temperature of the adsorption medium affects La(III) species (increases mobility of the La(III) ions and decreases the La(III) ions hydration sphere) and (ii) the temperature of the adsorption medium affects adsorbent characteristics (increases swelling and decreases the hydration sphere of the binding sites). These effects increase the penetration of La(III) ions inside the HA@ep@Ch and CM@HA@ep@Ch biosorbents, and hence increase the probability of interaction between La(III) and HA@ep@Ch or CM@HA@ep@Ch active sites. At all temperatures, the biosorption affinities of CM@HA@ep@Ch for La(III) is higher than those of HA@ep@Ch.

The effect of adsorbent dose (0.5–10.0 g/L) was investigated under optimum conditions of La(III) concentration (400 mg/L), temperature (300 K), and adsorption medium (50 mL). The bottles were shacked using a water bath shaker at 300.0 rpm. After 120 min, the biosorbents were filtrated, and the La(III) ions were determined in the filtrate. The biosorbent capacities (*q*_*e*_, mg/g) and biosorption efficiency (*q*_*e*_, %) were calculated using Eqs. 1 and 2. The biosorption capacities and efficiencies obtained at different adsorbent doses were plotted against solid/liquid ratio (g/L) (Fig. 7c). Increasing S/L ratio decreased biosorbent capacities (*q*_*e*_, mg/g), while it increased biosorption efficiency (*q*_*e*_, %). That is reasonably ascribed to an increase in the adsorbing surface area and adsorption sites with an increasing S/L ratio. According to Fig. 7c, the maximum La(III) biosorption efficiencies for HA@ep@Ch and CM@HA@ep@Ch are 89.2 and 93.8%, respectively.

### Biosorption isotherm and thermodynamic

**Fig. 8:**
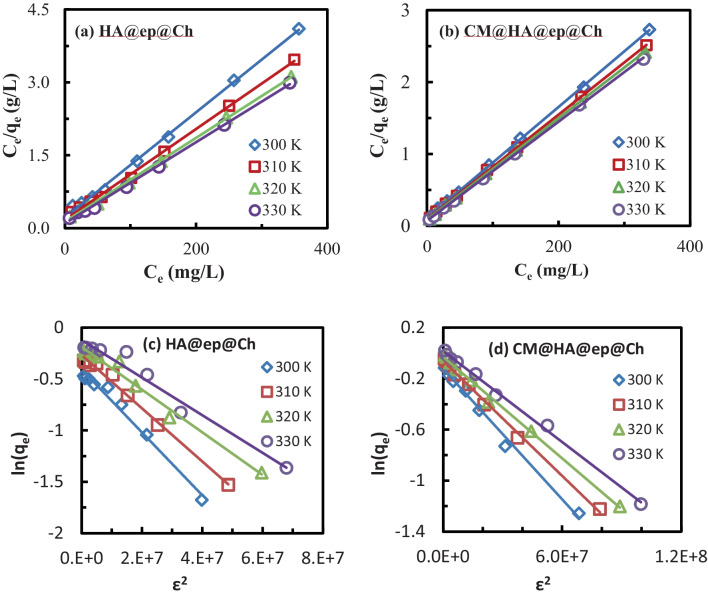
Langmuir (**a**, **b**) and D-R (**c** and **d**) isotherms for La(III) biosorption on HA@ep@Ch and CM@HA@ep@Ch at different temperatures

Various isotherm models were used to investigate the type and mechanism of La(III) adsorption on HA@ep@Ch and CM@HA@ep@Ch biosorbents. Table 2 lists the used isotherms models and their outcomes. According to the Langmuir model (Table 2), adsorption is a reversible monolayer process (Abd El-Magied et al. 2021b, Abd El-Magied 2016). The Langmuir adsorption capacity (*Q*_max_) and binding constant (*K*_*L*_) were obtained from the plots of Fig. 8 a and b. The outcomes of the model are presented in Table 2. The monolayer capacity increases with temperature, which may be due to increasing the probability of collisions between La(III) ions and the HA@ep@Ch or CM@HA@ep@Ch biosorbents surface as a result of increased mobility of the La(III) ions in the adsorption medium, along with increased dehydration of both La(III) species and the adsorbent surface. On the other hand, the value of *K*_*L*_ increases with temperature, indicating a binding enhancement between La(III) ions and the HA@ep@Ch or CM@HA@ep@Ch biosorbents surface at higher temperatures. At all temperatures, the sorption affinities of CM@HA@ep@Ch for La(III) are higher than those of HA@ep@Ch. The probability of multilayer adsorption of La(III) over HA@ep@Ch or CM@HA@ep@Ch was checked using the Freundlich model. The Freundlich isotherm describes the multilayer, non-ideal, reversible adsorption process (Abd El-Magied et al. 2021b, Abd El-Magied 2016). The results of La(III) adsorption at various temperatures (300–330 K) was analyzed by Freundlich model (Table 2) (SI, Fig. S2). The outcomes of the Freundlich model are presented in Table 2. At all temperatures and for both adsorbents, the slope of the plots is < unity, which indicates a favorable adsorption process (Fig. S4). Also, the Freundlich capacity (*K*_*F*_) of La(III) on CM@HA@ep@Ch is higher than that on HA@ep@Ch. However, the Freundlich capacity (*K*_*F*_) does not match the experimental capacity. In addition, the values of *R*^2^ of the Freundlich plots at different temperatures are less than those of the Langmuir plots obtained at the same conditions. This finding show that the that the Langmuir model is more accurate in describing the adsorption of La(III) on both HA@ep@Ch and CM@HA@ep@Ch adsorbents.

**Table 2:** A list of the used isotherms models and their outcomes

Model	Linear equation				Plot				
Langmuir	$\frac{{\textrm{C}}_{\textrm{e}}}{{\textrm{q}}_{\textrm{e}}}=\frac{{\textrm{C}}_{\textrm{e}}}{{\textrm{Q}}_{\textrm{max}}}+\frac{1}{{\textrm{K}}_{\textrm{L}}{\textrm{Q}}_{\textrm{max}}}$				C_e_/q_e_ versus C_e_				
Parameter	Definition	Value							
		HA@ep@Ch				CM@HA@ep@Ch			
T, K	Temperature	300	310	320	330	300	310	320	330
q_e_, mg/g	Experimental capacity	86.87	100.80	110.62	114.52	123.84	132.72	137.10	141.76
Q_max_, mg/g	Monolayer capacity	91.74	106.38	116.28	119.05	129.87	136.98	140.85	144.93
k_L_, L/mg	Binding constant	0.0510	0.0553	0.0650	0.0874	0.0728	0.0771	0.083	0.9801
k_L_, L/mol	Binding constant	7088.3	7685.2	9029.4	12141.6	10118.9	10707.6	11534.8	13614.3
R^2^	Correlation coefficient	0.9977	0.9978	0.9986	0.9981	0.9998	0.9997	0.9996	0.9998
Model	Linear equation				Plot				
Freundlich	$\log {\textrm{q}}_{\textrm{e}}=\log {\textrm{K}}_{\textrm{F}}+\frac{\log {\textrm{C}}_{\textrm{e}}}{\textrm{n}}$				logq_e_ versus logC_e_				
Parameter	Definition	Value							
		HA@ep@Ch				CM@HA@ep@Ch			
T, K	Temperature of the biosorption medium	300	310	320	330	300	310	320	330
K_f_, mg/g	Relative sorption capacity	8.96	19.47	23.75	27.40	31.22	33.91	36.40	39.70
n	Sorption intensity	2.25	3.17	3.38	3.55	3.79	3.83	3.93	4.07
R^2^	Correlation coefficient	0.8355	0.8094	0.8108	0.7596	0.8847	0.8905	0.8870	0.860
Model	Linear equation				Plot				
D-R	ln(q_e_) = ln(q_D_) − K_D_ε^2^ Where ε = Constant = RT ln[1+1/C_e_ ]				Ln(q_e_) versus ε^2^				
Parameter	Definition	Value							
		HA@ep@Ch				CM@HA@ep@Ch			
T, K	Temperature of the biosorption medium	300	310	320	330	300	310	320	330
q_D_, mg/g	Thioritical capacity of D-R	92.51	106.32	115.15	123.28	122.9	130.7	135.0	136.7
K_D_/1000	D-R binding constant	3.E-8	3.E-8	3.E-8	3.E-8	2.E-8	2.E-8	1.E-8	1.E-8
E, kJ/mol	The apparent energy	4.08	4.08	5.0	5.0	5.0	5.0	7.1	7.1
R^2^	Correlation coefficient	0.9824	0.9908	0.981	0.9646	0.9922	0.9956	0.9963	0.997

The Dubinin and Radushkevich (D-R) model describes the influence of the adsorbents’ porous structure on the adsorption process. It was based on an adsorption potential hypothesis that suggested that adsorption is connected to micropore volume filling rather than layer-by-layer adsorption on pores (Abd El-Magied et al. 2021b, Abd El-Magied 2016). The D-R isotherm model is better than the Langmuir isotherm model since it does not take into account a homogenous surface or a uniform distribution of energy. The D-R isotherm was used to investigate the type of La(III) interaction with HA@ep@Ch and CM@HA@ep@Ch adsorbents (as physical or chemical interaction). The adsorption data of La(III) interaction with HA@ep@Ch and CM@HA@ep@Ch adsorbents was treated with the D-R model (Table 2). Figure 8 c and d show the D-R plots of La(III) adsorption on HA@ep@Ch and CM@HA@ep@Ch adsorbents at different temperatures. The D-R modeling of the experimental data gives straight lines at the studied temperatures. The slop of the D-R plot equals (*β*), where the intercept equals (ln (*q*_*D*_)). The values of (*β*) were used to calculate the values of the apparent energy of the adsorption process (*E* (kJ/mol) = 1/√(2*K*_*D*_)). The outcomes of the D-R isotherm are presented in Table 2. The computed apparent energy of the adsorption process (*E*) values are 4.08–5 kJ/mol (for HA@ep@Ch) and 5–7.1 kJ/mol (for CM@HA@ep@Ch) (Table 2). These findings confirm the chemical nature of the La(III) interaction with HA@ep@Ch and CM@HA@ep@Ch adsorbents. The Van’t Hoff equation was used to investigate the nature of La(III) interaction with HA@ep@Ch and CM@HA@ep@Ch adsorbents through the outcomes of this equation (enthalpy (∆*H*°) and entropy (∆*S*°) change). These parameters are used to estimate the nature, spontaneity, and type of the reaction. The values of *K*_*L*_ obtained at 300–330 K (Langmuir model, Table 2) were used for the evaluation of thermodynamic parameters using the Van’t Hoff equation (Eq. 8) (Abd El-Magied et al. 2021a). The ln (*K*_*L*_, L mol^−1^) values were plotted against 1/temperature (1/*T*, 1/K), SI, Fig. S5.

8 $$\mathrm{ln}{K}_{L}=\frac{\Delta {S}^{^\circ }}{R}-\frac{{\Delta H}^{^\circ }}{RT}$$

where *R* (8.314 J/K mol) is the gas constant. Table 3 lists the outcomes of the Van’t Hoff equation. The values of ∆*H*° are positive values at all studied temperatures, confirming the endothermic nature of the La(III) interaction with the HA@ep@Ch and CM@HA@ep@Ch adsorbents. Also, the values of ∆*S*° are positive values at all studied temperatures, confirming the high affinity of the HA@ep@Ch and CM@HA@ep@Ch adsorbents for La(III) ions and referring to the increasing randomness at the solid/solution interface during the biosorption process. The values of ∆*H*° and ∆*S*° were used to calculate the ∆*G*° (Gibbs free energy) of the La(III) interaction with the HA@ep@Ch and CM@HA@ep@Ch adsorbents (Lima et al. 2019).

**Table 3:** Thermodynamic parameters

Adsorbents	Temp. (Kelvin)	∆*H°* (kJ/mol)	∆*S*° (J/mol K)	*T*∆*S*° (J/mol)	∆*G*° (J/mol)
HA@ep@Ch	300 310 320 330	14,494.63	124.2943	37,288.29 38,531.233 39,774.176 41,017.119	− 22,793.6624 − 24,036.6054 − 25,279.5484 − 26,522.4914
CM@HA@ep@Ch	300 310 320 330	7877.598	102.7195	30,815.841 31,843.0357 32,870.2304 33,897.4251	− 22,938.24286 − 23,965.43756 − 24,992.63226 − 26,019.82696

9 $$\Delta {G}^{\mathrm{o}}=\Delta {H}^{\mathrm{o}}-T\Delta {S}^{\mathrm{o}}$$

The obtained values of ∆*G*° are negative values at all studied temperatures, confirming the high affinity and spontaneity of the HA@ep@Ch and CM@HA@ep@Ch interaction with La(III) ions. The obtained values of ∆*G*° refer to the increasing randomness at the solid/solution interface during the biosorption process. The increase in ∆*G*° values with temperature implies that the adsorption reaction is more spontaneous at high temperatures (Abd El-Magied et al. 2021a). This may be related to increasing the mobility of La(III) ions at higher temperatures, causing the probability of La(III) collusion with the adsorbent to increase.

### La(III) elution and biosorbent regeneration for the repeated use

The elution of La(III) from the loaded biosorbents was studied using different concentrations of hydrochloric acid (from 0.01 to 0.5 M HCl). A 0.5-M HCl solution was found effective to elute 95.8% of the adsorbed lanthanum on HA@ep@Ch, and 93.4% of the adsorbed lanthanum on CM@HA@ep@Ch. The eluted biosorbents were regenerated by washing and then soaking into water for 24 h. The regenerated biosorbents were reused for capture La(III) ions from fresh La(III) solution (second run). The biosorbent reusability was evaluated through three consecutive adsorption/elution cycles. After theses three times repetitions, the sorption capacity of HA@ep@Ch and CM@HA@ep@Ch were 90–93% of the corresponding values of the first run.

### Biosorption of La(III) from the monazite leach liquor

The most important primary source of rare earth elements is monazite. Monazite is an anhydrous orthophosphate mineral that occurs as an accessory mineral in certain vein deposits and in acidic igneous and metamorphic rocks. The monazite sample was concentrated from the black sand raw material through successive separation and concentration processes. The monazite sample was firstly processed magnetically to exclude the paramagnetic minerals (e.g., magnetite, ilmenite, and leucoxene) particularly they form the majority of the black sand. The free-paramagnetic portion was treated electrostatically by which two portions were obtained; conductor and non-conductor fractions. The non-conductor part was finally exposed to fractionation using wet-table separation where the tail-table strip and top-table strip resulted. The top-table strip was dried and microscopically investigated where a high-grade monazite concentrate was obtained. Ten grams were taken through careful quartering of the high-grade monazite concentrate and exposed for well grinding to conduct the grain size less than 0.063 mm. For the analysis of the major components, 0.5 g of the ground sample was completely dissolved using acid mixture (HF + H_2_SO_4_ + HNO_3_) under heating (200–250 °C). Digestion was carried out till mushy. The mashed sample was then acidified with 20 mL of HCI (1: 1), and then heated for 1 h. The acidified sample volume was diluted to a total volume of 0.25 L with water (de-ionized). SiO_2_ and Al_2_O_3_ contents were determined in a dissolve monazite solution produced by alkaline digestion. In detail, the monazite sample was fused with NaOH in a nickel crucible. After cooling, the fused sample was dissolved in 1:1 HC1 solution under heating, and then was transferred into measuring flask, and its volume was completed to 250 mL with de-ionized water to obtain the sample solution ready to be used for SiO_2_ and Al_2_O_3_ determination. The types and concentrations of the major constituents were chemically spectrophotometrically quantified and the resultants are shown in the Table 4. The types and concentrations of the trace constituents were achieved through the chemical analysis using ICP-OES.

**Table 4:** Chemical analysis of the monazite concentrate

Constituents	Concentration	RE_2_O_3_ constituents	
	Wt, %	Constituents	Concentration, %
REE_2_O_3_	52.66	La_2_O_3_	12.32
ThO_2_	6.12	CeO_2_	23.38
U_3_O_8_	0.37	Nd_2_O_3_	10.11
P_2_O_5_	27.24	Pr_2_O_3_	2.65
TiO_2_	2.93	Sm_2_O_3_	1.93
SiO_2_	1.32	Gd_2_O_3_	0.98
Fe_2_O_3_	2.89	Y_2_O_3_	1.29

Lanthanum recovery from monazite concentrate was achieved by two successive separation steps, i.e., leaching followed by extraction by adsorption on HA@ep@Ch and CM@HA@ep@Ch biosorbents. The monazite chemical leaching with achieved by acidic digestion of monazite with hot sulfuric acid. The monazite sample was taken through careful quartering from the high-grade monazite concentrate and exposed for well grinding to conduct the grain size less than 0.063 mm (− 200 mesh). The monazite sample was added portionwise to hot sulfuric acid (185 °C, 98%) under mechanical stirring. The reaction mixture was stirred for 4 h at 225 °C. The reaction slurry (U, Th, and REE sulfates) was allow to cool; then, 4 L of ice water was added to it under stirring for 30 min. The reaction mixture was filtered, and the lanthanum content into the obtained monazite leach liquor was measured. The results show that the obtained monazite leach liquor contains 148.21 mg/L of lanthanum ions. The obtained monazite leach liquor was treated with the HA@ep@Ch and CM@HA@ep@Ch adsorbents for lanthanum recovery. For lanthanum recovery, 0.3 g of the HA@ep@Ch and CM@HA@ep@Ch was added to a flask containing 25 mL of monazite leach liquor. The flasks were shaken (250 rpm) at 300 K for 30 min. After equilibration, HA@ep@Ch and CM@HA@ep@Ch were filtered, and the remaining concentration of La(III) into the liquor was measured, where adsorption capacity (mg/g) and efficiency (%) values were calculated. The obtained results show that about 70.03 and 74.63% of the lanthanum content was adsorbed by HA@ep@Ch and CM@HA@ep@Ch, respectively. These results show (Fig. S6) that lanthanum ions were recovered effectively from monazite leach liquor using the HA@ep@Ch and CM@HA@ep@Ch adsorbents. The adsorbents showed greater selectivity in extracting La, Ce, Pr, Nd, and Sm (62–75%) from REE leach liquid compared to extracting other REEs (20–41%). So, the adsorbents are very effective in the recovery of lanthanum from REE leach liquor (monazite). Table 5 compares the ability of HA@ep@Ch and CM@HA@ep@Ch adsorbents to absorb lanthanum ions with other adsorbents in some recently published articles. This comparison shows that the HA@ep@Ch and CM@HA@ep@Ch adsorbents used in this study are promising adsorbents in the field of lanthanum extraction.

**Table 5:** The ability of HA@ep@Ch and CM@HA@ep@Ch to adsorb lanthanum ions compared to other adsorbents in some recently published articles

Adsorbents	pH	*q*_*m*_, mg/g	Reference
Carboxylated cellulose	6	33.7	Li et al. (2020)
DowexTM M4195 Lewatit® TP 207	2	9.9 22.6	Botelho Junior et al. (2022)
La-IIP-Schiff base La-IIP-Azo	6	25.0 24.3	Nik Mustapa et al. (2016)
Pinus brutia leaf powder	5	22.9	Kütahyali et al. (2010)
Pristine	5	49.5	
Sodium alginate/goethite	5	64–78	Sayed et al. (2021)
DTPA@magnetic NPs	6	63.1	Almeida and Toma (2016)
Durian rind	5	71.4	Kusrini et al. (2019)
Poly(6-acryloylamino-hexyl hydroxamic acid) Resin	1.5	143.1	Cao et al. (2020)
HA@ep@Ch	6	114.52	*Here*
CM@HA@ep@Ch	6	141.76	*Here*

## Conclusion

In this study, chitosan (Ch) with a significant degree of deacetylation, purity, and solubility was produced from shrimp waste, and then, it was modified with NH_2_ and COOH ligands to produce different functionalized cross-linked chitosan/poly-amine (HA@ep@Ch) and poly carboxylated/imine chitosan (CM@HA@ep@Ch) biosorbents. The yield of chitin extracted from shrimp waste is 21.1%. The yield of the shrimp chitosan is 17.21. Shrimp waste, chitin, and chitosan have moisture contents of 68.5, 8.1, and 9.56%, respectively. ep@Ch, HA@ep@Ch, and CM@HA@ep@Ch have moisture contents of 9.21, 9.95, and 9.15, respectively. The obtained shrimp chitosan had 0.21% ash content. The ep@Ch, HA@ep@Ch, and CM@HA@ep@Ch have ash contents of 0.22, 0.23, and 0.24, respectively. The recovery of lanthanum by the modified chitosan adsorbents was thoroughly explored and discussed. The results obtained indicate that the quantity of adsorption rises as the pH of the medium rises until the medium pH reaches 6, after which the increase in the amount of adsorption is almost imperceptible and can be ignored. The results obtained indicate that the amount of adsorption increases with the increase in the duration time (the biosorption rate raised rapidly) and in La(III) concentration until equilibrium is attained. This study demonstrates that the La(III) uptake on HA@ep@Ch and CM@HA@ep@Ch is dictated by pore-diffusion control. The results show that the Langmuir isotherm and Dubinin and Radushkevich models are more accurate in describing the biosorption of La(III) on both HA@ep@Ch and CM@HA@ep@Ch adsorbents. The adsorbents were very effective in the recovery of lanthanum from REE leach liquor. The suggested modes of interaction between La(III) species and the biosorbents are complexation and cation exchange reactions.

### Supplementary Information

Below is the link to the electronic supplementary material.

Supplementary file1 (DOCX 1079 KB)

## Data Availability

All data generated or analyzed during this study are included in this published article (and its supplementary information files).
