# Supplementary material for: Extraction of rare earth elements from monazite leach liquor using functionalized chitosan sorbents derived from shrimp waste
Source: Environ Sci Pollut Res Int. 2023 Sep 25;30(49):108067–84. doi: 10.1007/s11356-023-29662-8 (PMC10611849; doi:10.1007/s11356-023-29662-8)
Supplement: Supplementary file 1 — Supplementary file1 (DOCX 1079 KB) [file 11356_2023_29662_MOESM1_ESM.docx]

**Supplementary information (SI)**

**
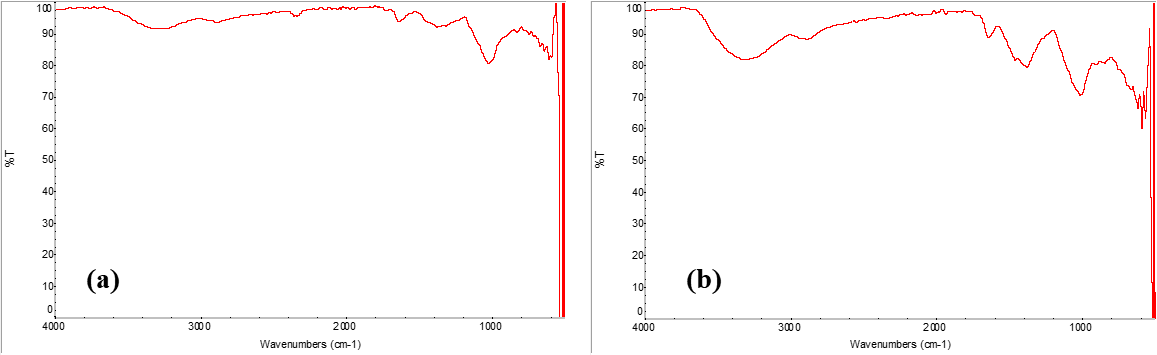
**

**Fig. S1.** FT-IR spectra of HA@ep@Ch (a), and CM@HA@ep@Ch (b) sorbents

**
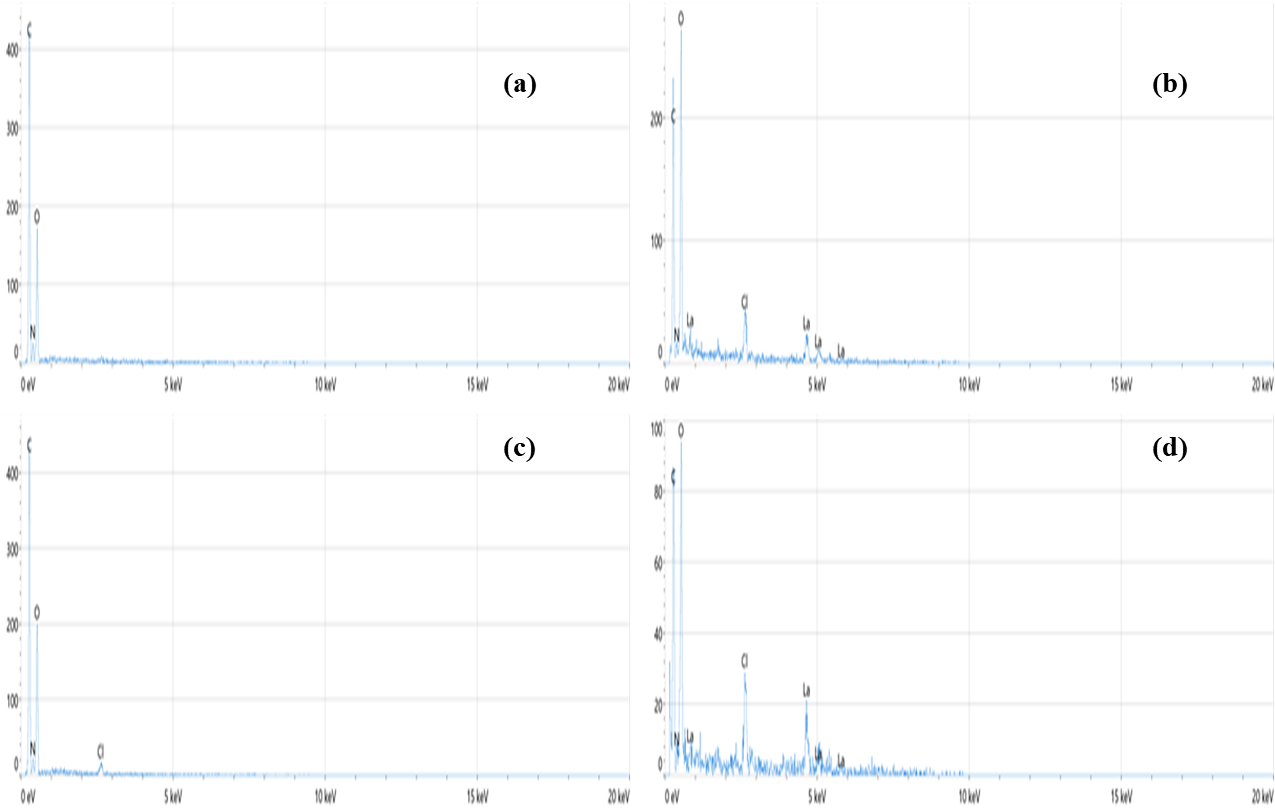
**

**Fig. S2** EDX charts of HA@ep@Ch (a), La (III)-loaded HA@ep@Ch (b), CM@HA@ep@Ch (c), and La (III)-loaded CM@HA@ep@Ch (d) sorbents

**Fig. S3**. Effect of ionic strength on La(III) biosorption by HA@ep@Ch and CM@HA@ep@Ch sorbents

**Fig. S4.** Freundlich isotherm for La(III) biosorption on HA@ep@Ch and CM@HA@ep@Ch sorbents at different temperatures

**Fig. S5**. Van't Hoff equation for La(III) biosorption on HA@ep@Ch and CM@HA@ep@Ch sorbents

**Fig. S6**. REE extraction from the monazite leach liquor using the HA@ep@Ch and CM@HA@ep@Ch adsorbents
